# Comparative study of the pharmacokinetics, efficacy and safety of ET-26 in elderly and non-elderly subjects: the results of a phase I clinical trial

**DOI:** 10.3389/fphar.2025.1665322

**Published:** 2025-10-16

**Authors:** Fan Yang, Jing Sun, Pan-Pan Ye, Wen-Shuo Lv, Li-Ze Li, Bao-Zhong Zhao, John Van Den Anker, Yi Zheng, Bo-Wen Ke, Xiao-Ran Yang, Wei Zhao

**Affiliations:** ^1^ Department of Clinical Pharmacy, Key Laboratory of Chemical Biology (Ministry of Education), School of Pharmaceutical Sciences, Cheeloo College of Medicine, Shandong University, Jinan, China; ^2^ Avanc Pharmaceutical Co., Ltd., Jinzhou, China; ^3^ Division of Clinical Pharmacology, Children’s National Hospital, Washington, DC, United States; ^4^ Departments of Pediatrics, Pharmacology & Physiology, Genomics & Precision Medicine, School of Medicine and Health Sciences, George Washington University, Washington, DC, United States; ^5^ Department of Paediatric Pharmacology and Pharmacometrics, University Children’s Hospital Basel, University of Basel, Basel, Switzerland; ^6^ Department of Anesthesiology, Laboratory of Anesthesia and Critical Care Medicine, National-Local Joint Engineering Research Centre of Translational Medicine of Anesthesiology, West China Hospital, Sichuan University, Chengdu, China

**Keywords:** methoxyethyl etomidate hydrochloride, elderly, pharmacokinetics, safety, efficacy

## Abstract

**Aim:**

To evaluate the pharmacokinetics, pharmacodynamics, and safety of the novel systemic intravenous anesthetic ET-26—an etomidate derivative designed to reduce adrenal suppression—in healthy elderly and non-elderly subjects.

**Methods:**

In this Phase I, single-center, non-randomized, open-label trial, 16 volunteers were enrolled: eight elderly (≥65 years, including ≥75 years) and eight non-elderly (18–64 years), matched for gender and body weight. Each received a standardized IV infusion of ET-26. Plasma concentrations were measured for plasma protein binding, C_max,_ and AUC; time to loss of consciousness (LOC) and safety were assessed.

**Results:**

In 16 subjects (8 elderly/8 non-elderly), ET-26 showed higher exposure in the elderly (C_max_ GMR 198.81%, 90% CI 126.51–312.45) and ${\text{AUC}}_{0-\infty }$ was 23.5% higher (90% CI: 107.6%–141.9%) with comparable pharmacodynamics (median LOC time 1.933 min). Plasma protein binding remained stable (intergroup difference ≤1.0%). Drug-related TEAEs (37.5%) were mild and self-limiting.

**Conclusion:**

Despite elevated systemic exposure in elderly subjects, ET-26 demonstrates comparable efficacy and retains a favorable tolerability profile across age groups, eliminating the need for dose adjustments in elderly populations.

**Clinical Trial Registration:**

http://www.chinadrugtrials.org.cn/clinicaltrials.searchlist.dhtml?keywords=CTR20233784, identifier CTR20233784.

## Background

Methoxyethyl etomidate hydrochloride (ET-26) is a novel intravenous general anesthetic developed analog of etomidate. By incorporating a methoxyethyl substituent, ET-26 is rapidly hydrolyzed by nonspecific esterases, resulting in a markedly shorter context-sensitive half-time (Wang et al., 2017b; Jiang et al., 2024). This structural modification enables ET-26 to retain the rapid-onset hypnotic action and hemodynamic stability of etomidate while significantly reducing its inhibition of adrenal 11β-hydroxylase, especially in elderly patients who often exhibit decreased hepatic and renal clearance (Wang et al., 2017a; Wang et al., 2017b). In preclinical evaluations, ET-26 demonstrated favorable pharmacokinetic (PK) characteristics and safety in aged rats, with its rapid metabolism profile suggesting potential clinical applicability in elderly populations (Chang et al., 2022). Phase I clinical studies employing both single ascending dose and multiple dosing regimens have demonstrated that ET-26 is safe and well tolerated in healthy subjects at doses up to 2.8 mg·kg^−1^. Moreover, compared with etomidate at 0.3 mg·kg^−1^, ET-26 exhibits superior preservation of adrenal cortical function (Yin et al., 2025). A Phase III clinical trial (NCT06203431, registered 11 October 2023.) is currently underway to evaluate the efficacy and safety of ET-26 for the induction of general anesthesia in elective surgical patients aged 18–70 years, with recruitment ongoing.

Currently, research on ET-26 is primarily focused on healthy adult subjects and animal models, while the pharmacokinetic characteristics in elderly patients remain unclear (Chang et al., 2022). With the increasing prominence of global aging issues and the inevitable physiological changes that accompany aging, including decreased organ function, altered body composition, and changes in receptor sensitivity, the processes of drug absorption, distribution, metabolism, and elimination can be significantly affected (Hammerlein et al., 1998; Shafer, 2000; Mangoni and Jackson, 2004; Curtis et al., 2005; Yizhak et al., 2013). For instance, reduced hepatic blood flow and decreased glomerular filtration rate in the elderly may lead to diminished drug clearance, a prolonged elimination half-life, and an increased risk of adverse drug reactions (Strom et al., 2016; Andres et al., 2019). Therefore, it is essential to establish the pharmacokinetic profile, efficacy, and safety of ET-26 in healthy elderly individuals and to provide precise dosing recommendations based on the specific pharmacological characteristics of this population.

## Methods

### Study design and population

This single-center, non-randomized, open-label Phase I clinical trial was designed to evaluate the pharmacokinetic (PK) and pharmacodynamic (PD) profiles of ET-26 in healthy subjects, comprising both non-elderly and elderly populations. A total of 16 healthy subjects were enrolled, including 8 non-elderly subjects aged 18–64 years and 8 elderly subjects aged ≥65 years, with at least 3 subjects being ≥75 years old. To ensure group comparability, the non-elderly group was matched to the elderly group in terms of gender, and the mean body weight of non-elderly subjects was maintained within ±10 kg of the elderly group’s mean. All participants provided informed consent before participation in the trial. This study had appropriate inclusion/exclusion criteria and was approved by the Ethics Committee of the Shandong Provincial Qianfoshan Hospital before implementation (Supplementary Material S1).

### Pharmacokinetic assessment

Plasma concentrations of ET-26 at various time points were measured using a validated liquid chromatography-tandem mass spectrometry (LC-MS/MS) method. Pharmacokinetic samples were collected according to the planned schedule (Supplementary Table S1), and the bioanalytical methods are provided in the Supplementary Material S1.

Data from both the elderly and non-elderly groups were analyzed to generate individual as well as mean (Mean ± SD) plasma concentration-time (c-t) curves and semi-logarithmic c-t curves. The key PK parameters of ET-26 include Peak concentration (C_max_), Time to peak concentration (T_max_), Area under the concentration-time curve (AUC_0-t_, ${\text{AUC}}_{0-\infty }$ ), Percentage extrapolated AUC (AUC__%Extrap_), Elimination half-life (T_1/2_), Apparent clearance (CL, CL/F), Apparent volume of distribution (Vz, Vz/F), and Mean residence time (MRT) were calculated via a non-compartmental model using WinNonlin software (version 8.1), based on the actual sampling times and administered dose. For each group, the arithmetic mean, standard deviation, and coefficient of variation were computed. Descriptive statistical summaries of ET-26 plasma protein binding data (including mean ± standard deviation) were generated using SAS software.

After the log transformation of the primary PK parameters (C_max_, AUC_0-t_, and ${\text{AUC}}_{0-\infty }$ ), analysis of variance and two-sided tests were performed to calculate the geometric mean ratios and 90% confidence intervals. If the 90% confidence intervals fall within the 80.0%–125.0% range, it is concluded that there is no significant difference in the PK profiles between the non-elderly and elderly groups; otherwise, a comprehensive evaluation, incorporating exposure-response relationships, will be conducted to guide clinical dosing in the elderly population.

### Pharmacodynamic assessment

Following the Technical Guidelines for Clinical Evaluation of Intravenous General Anesthetics (NMPA, 2022), the pharmacodynamic evaluation in this study focused on two primary endpoints: the time to loss of consciousness and the area under the curve (AUC) of the Bispectral Index (BIS). Loss of consciousness was assessed by continuously monitoring the Modified Observer’s Assessment of Alertness/Sedation (MOAA/S) scale, with the endpoint defined as the interval from administration to the first instance when the MOAA/S score reached ≤1. Simultaneously, BIS values were recorded via electroencephalography to determine the minimum BIS level. The resulting BIS curves were subsequently fitted, and the AUC was calculated using WinNonlin or SAS 9.4 software. Descriptive statistics, including mean, median, standard deviation, maximum, and minimum values, were then used to comprehensively characterize the anesthetic effect.

### Safety analysis

Descriptive statistical methods were employed to evaluate laboratory test results, vital signs, 12-lead electrocardiograms (ECG), physical examination findings, and additional safety parameters. Adverse events (AEs) were coded per the International Council for Harmonisation (ICH) Medical Dictionary for Regulatory Activities (MedDRA, version 25.1) and their severity was graded according to the National Cancer Institute’s Common Terminology Criteria for Adverse Events (CTCAE, version 5.0) (see Supplementary Table S2). Treatment-emergent adverse events (TEAEs), serious adverse events (SAEs), and TEAEs leading to study discontinuation were systematically assessed. These events were further categorized based on participant subgroup (elderly vs. non-elderly), severity, and their causality relative to the investigational drug.

## Results

### Demographic characteristics

A total of 16 subjects were enrolled in the study, with 8 allocated to the healthy elderly group and 8 to the healthy non-elderly group. The median age was 57.5 years (range: 24–81 years). All subjects were male and of Han ethnicity. The median height was 164.75 cm (range: 153.0–173.5 cm), the median weight was 63.8 kg (range: 56.1–81.5 kg), and the median body mass index (BMI) was 23.15 kg/m^2^ (range: 19.3–27.7 kg/m^2^) (Table 1).

**TABLE 1:** Summary of demographics and subject characteristics.

Categorical variable	Total	Healthy elderly group	Healthy non-elderly group
	N (%)	N (%)	N (%)
Sex			
Male	16 (100%)	8 (50%)	8 (50%)
Female	-	-	-
Ethnicity			
Han	16 (100%)	8 (100%)	8 (100%)
Continuous variable	Median (range)	Median (range)	Median (range)
Age (y)	57.5 (24–81)	71.0 (65–81)	30.0 (24–50)
Body height (cm)	164.75 (153.0–173.5)	164.50 (153.0–173.0)	167.50 (163.5–173.5)
Body weight (kg)	63.8 (56.1–81.5)	64.65 (56.5–81.5)	63.80 (56.1–69.2)
Body mass index (kg/m^2^)	23.15 (19.3–27.7)	24.95 (22.1–27.7)	23.00 (19.3–24.9)

### Plasma protein binding rate/free fraction

After intravenous administration of 0.8 mg/kg ET-26, plasma protein binding of ET-26 and the free fraction showed no significant time-dependent changes in healthy elderly subjects (n = 8) and healthy non-elderly subjects (n = 8). In the elderly group, plasma protein binding was 56.3% (free fraction 0.437) at 1 min post - dose, showing a slight increase to 60.3% (free fraction 0.397) after 4 h. In the non-elderly group, the binding rate was 57.3% (free fraction 0.427) at 1 min and 61.0% (free fraction 0.390) at 4 h. The binding rate fluctuated slightly within 4 h in both groups (4.0% in the elderly, 3.7% in the non-elderly), with no regular upward or downward trend. The inter-group comparison showed that the plasma protein binding difference was within 1.0% (1.0% at 1 min, 0.7% at 4 h), and the free fraction difference was less than 0.04 units at corresponding time points.

### Pharmacokinetic analyses

**FIGURE 1:**
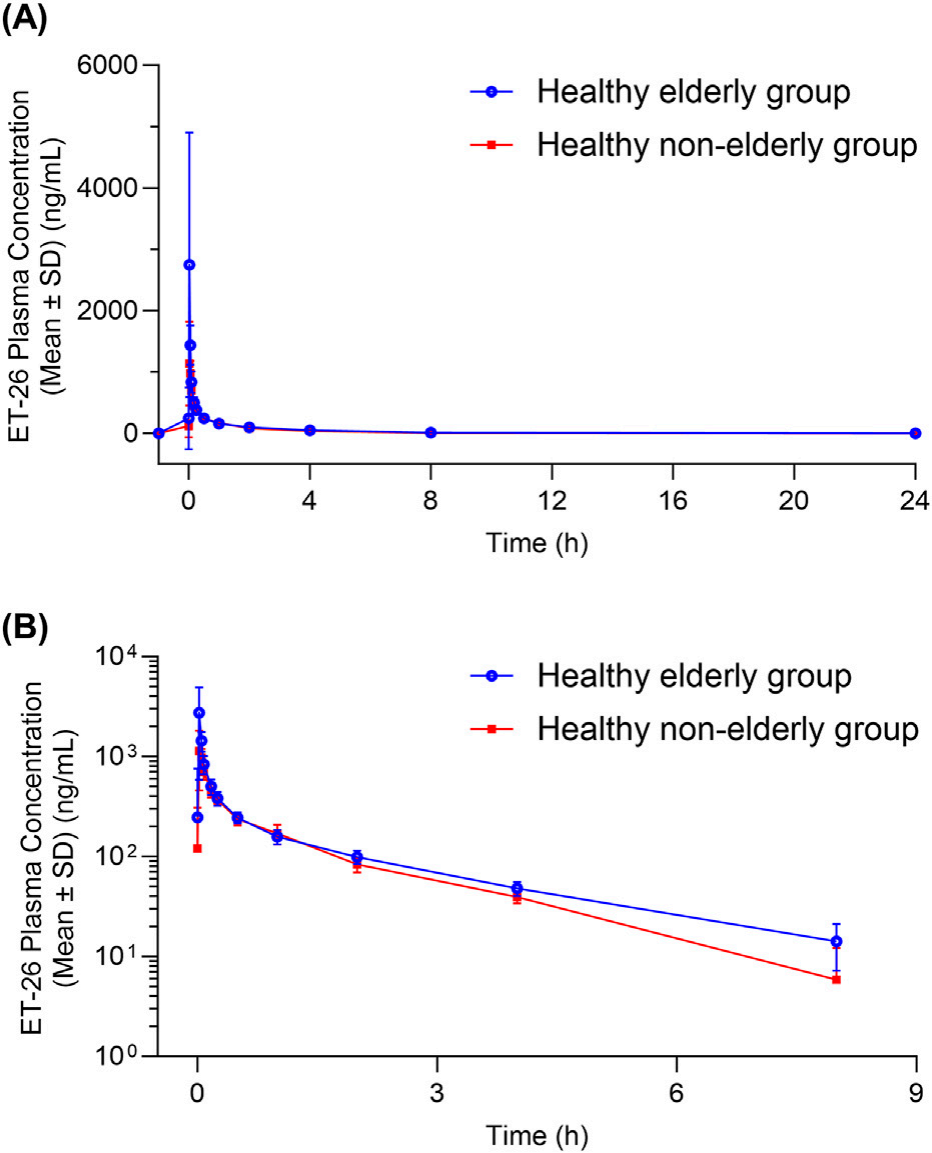
Plasma Concentration-Time curves of ET-26 in Healthy Elderly and Non-Elderly Subjects **(A)** Linear plot and **(B)** semi-logarithmic plot. The blue line with blue circles represents healthy elderly subjects, while the red line with red squares represents healthy non-elderly subjects.

Eight subjects in the healthy elderly group received a single intravenous injection of ET-26 at a dose of 0.8 mg/kg. The median T_max_ was 1.94 min. Geometric mean PK parameters were as follows: C_max_ of 2,500 ng/mL, AUC_0-t_ of 804 h·ng/mL, ${\text{AUC}}_{0-\infty }$ of 865 h·ng/mL. Eight subjects in the healthy non-elderly group received the identical dosing regimen. The median T_max_ was 4.00 min. Their geometric mean PK parameters were: C_max_ of 1,260 ng/mL, AUC_0-t_ of 641 h·ng/mL, and ${\text{AUC}}_{0-\infty }$ of 700 h·ng/mL. Comparing the two groups (n = 16), the geometric mean ratio for C_max_ (elderly vs. non-elderly) was 198.81% (90% CI: 126.51–312.45). The corresponding ratios for AUC_0-t_ and ${\text{AUC}}_{0-\infty }$ were 125.43% (90% CI: 107.56–146.27) and 123.54% (90% CI: 107.57–141.87), respectively. Since these confidence intervals do not entirely fall within the predefined equivalence range of 80%–125% (with overlap at the boundaries), further evaluation incorporating the exposure-response relationship is warranted (Table 2; Figure 1).

**TABLE 2:** The plasma pharmacokinetic parameters of ET-26 in elderly and non-elderly subjects.

Parameter	Healthy elderly group			Healthy non-elderly group			GMR (%)	(90% IC)
	Mean	SD	CV%	Mean	SD	CV%		
T_max_ (min)	2.48	0.94	38.07	3.23	1.07	33.17		
C_max_ (ng/mL)	2,970	1880	63.35	1,330	529	39.72	198.81	(126.51–312.45)
AUC_0-t_ (h·ng/mL)	816	144	17.62	648	100	15.46	125.43	(107.56–146.27)
${\text{AUC}}_{0-\infty }$ (h·ng/mL)	879	155	17.63	704	78.2	11.1	123.54	(107.57–141.87)
AUC __% Exmp_ (%)	7.04	3.06	43.44	8.38	4.7	56.06		
T_1/2_ (h)	2.21	0.1	8.46	1.81	0.407	22.48		
CL (L/h)	61.3	11.7	19.12	71.1	7.12	10.01		
Vz (L)	196	43.4	22.11	184	34.8	18.95		
MRT_0-t_ (h)	1.55	0.3	19.16	1.32	0.31	23.1		
${\text{MRT}}_{0-\infty }$ (h)	2.17	0.27	12.35	1.86	0.3	15.98		

T_max_, time to reach maximum plasma concentration; C_max_, maximum plasma concentration; AUC_0-t_, area under the plasma concentration-time curve from time zero to the last measurable concentration; ${\text{AUC}}_{0-\infty }$ , area under the plasma concentration-time curve from time zero to infinity; AUC__% Exmp_, residual area percent $\text{AUC}{\_}_{\%\;\text{Extrap}}=\left(\left[{\text{AUC}}_{0-\infty }-{\text{AUC}}_{0-t}\right]/{\text{AUC}}_{0-\infty }\right)\times 100\%$ ; T_1/2_, terminal half-life, the time for the plasma concentration to decrease by half; CL, total plasma clearance, representing the volume of plasma from which the drug is completely removed per unit of time; Vz, apparent volume of distribution at steady state, reflecting the volume in which the drug would be uniformly distributed to achieve the observed plasma concentration; MRT_0-t_, mean residence time from time zero to the last measurable concentration; ${\text{MRT}}_{0-\infty }$ , mean residence time from time zero to infinity, representing the average time the drug remains in the body. Mean, arithmetic mean; SD, standard deviation; CV, coefficient of variation; GMR, geometric mean ratio.

### Pharmacodynamic analysis

In the healthy elderly group, the median time to loss of consciousness post-dose was 1.933 min (range: 0.93–2.93 min). The area under the BIS curve was 855.55 (range: 711.89–1,345.71) (Table 3).

**TABLE 3:** Summary of duration of absence of consciousness and BIS score.

Parameter	Healthy elderly group	Healthy non-elderly group	Total
	N = 8	N = 8	N = 16
Time to loss of consciousness (min)			
N (%)	8 (100.0)	7 (87.5)	15 (93.8)
Mean (SD)	1.82 (0.64)	1.933 (0.02)	1.870 (0.46)
Median	1.933	1.933	1.933
Range	(0.93–2.93)	(1.92–1.97)	(0.93–2.93)
BIS area under the curve (AUC)			
N (%)	8 (100.0)	8 (100.0)	16 (100.0)
Mean (SD)	916.41 (204.88)	619.796 (181.16)	768.10 (241.59)
Median	855.55	648.68	767.40
Range	(711.89–1,345.71)	(240.67–816.13)	(240.67–1,345.71)

Mean, arithmetic mean; SD, standard deviation.

In the healthy non-elderly group, one subject exhibited a MOAA/S score >1 due to a response to repeated loud calls of his name. Excluding this subject, the remaining 7 subjects showed a median loss of consciousness time of 1.933 min (range: 1.92–1.97 min), with a BIS AUC of 648.68 (range: 240.67–816.13).

### Safety outcomes

In this study, 9 TEAE events were reported in 6 subjects. Among these, 7 events were deemed related to the study drug, resulting in an overall incidence of 37.5% (6/16 subjects). In the healthy elderly group, drug-related TEAEs comprised Grade 1 injection site pain (12.5%, 1/8) and Grade 2 cough (12.5%, 1/8). The cough was managed with non-pharmacological treatment (suctioning), and no treatment was required for the other events; all recovered completely. In contrast, in the healthy non-elderly group, drug-related TEAEs included Grade 1 injection site pain (50.0%, 4/8) and myoclonus (12.5%, 1/8), none of which required any pharmacological or non-pharmacological intervention, and all recovered (Table 4).

**TABLE 4:** Listing of treatment-emergent adverse events.

TEAEs	Healthy elderly group		Healthy non-elderly group		Total	
	N=8		N=8		N=16	
	N (%)	Case	N (%)	Case	N (%)	Case
Systemic Disorders and Administration Site Conditions	1 (12.5)	1	4 (50.0)	4	5 (31.3)	5
Grade 1	1 (12.5)	1	4 (50.0)	4	5 (31.3)	5
Injection site pain	1 (12.5)	1	4 (50.0)	4	5 (31.3)	5
Grade 1	1 (12.5)	1	4 (50.0)	4	5 (31.3)	5
Investigations	1 (12.5)	1	0 (0.0)	0	1 (6.3)	1
Grade 1	1 (12.5)	1	0 (0.0)	0	1 (6.3)	1
Increased blood glucose	1 (12.5)	1	0 (0.0)	0	1 (6.3)	1
Grade 1	1 (12.5)	1	0 (0.0)	0	1 (6.3)	1
Nervous System Disorders	0 (0.0)	0	1 (12.5)	1	1 (6.3)	1
Grade 1	0 (0.0)	0	1 (12.5)	1	1 (6.3)	1
Myoclonus	0 (0.0)	0	1 (12.5)	1	1 (6.3)	1
Grade 1	0 (0.0)	0	1 (12.5)	1	1 (6.3)	1
Respiratory, Thoracic, and Mediastinal Disorders	1 (12.5)	1	0 (0.0)	0	1 (6.3)	1
Grade 2	1 (12.5)	1	0 (0.0)	0	1 (6.3)	1
Cough	1 (12.5)	1	0 (0.0)	0	1 (6.3)	1
Grade 2	1 (12.5)	1	0 (0.0)	0	1 (6.3)	1
Cardiac Disorders	1 (12.5)	1	0 (0.0)	0	1 (6.3)	1
Grade 1	1 (12.5)	1	0 (0.0)	0	1 (6.3)	1
Ventricular extrasystole	1 (12.5)	1	0 (0.0)	0	1 (6.3)	1
Grade 1	1 (12.5)	1	0 (0.0)	0	1 (6.3)	1

TEAEs, treatment-emergent adverse events.

## Discussion

Elderly patients often present with evident physiological changes, including reduced hepatic and renal clearance and decreased plasma protein binding (PPB), which may affect the pharmacokinetics and pharmacodynamics of ET-26 (Sadean and Glass, 2003; Schlender et al., 2016; Thurmann, 2020). Considering the age-related challenges posed by the elderly, it is essential to investigate the PK, efficacy, and safety profile of ET-26 in this population. This study enrolled 16 subjects, with 8 in the healthy elderly group and 8 in the healthy non-elderly group. Demographic characteristics were consistent across groups in terms of gender and ethnicity.

In our study, ET-26 exhibited moderate plasma protein binding of 56.3%–60.3% in elderly subjects and 57.3%–61.0% in non-elderly subjects over a 4-h post-dose interval, with free fractions of approximately 0.397–0.437 and minimal time-dependent variation (<4.0% in elderly vs. 3.7% in non-elderly). These findings indicate that age has no appreciable effect on ET-26 PPB, despite well-documented age-related changes in plasma protein levels. Compared to etomidate (75% PPB) (Valk and Struys, 2021), ET-26 exhibited a high plasma protein binding rate, with minimal fluctuations in binding rates and free fractions observed in both groups throughout the monitoring period, indicating relatively stable plasma protein binding characteristics.

The pharmacokinetic analysis demonstrated that following a 1-min intravenous infusion of ET-26, healthy elderly subjects exhibited a geometric mean C_max_ that was 198.81% (90% CI: 126.51%–312.45%) of that observed in healthy non-elderly subjects. In the elderly group (n = 8), five participants reached T_max_ between 1.92 and 1.95 min, one at 2.15 min, and two at 4.00 min. By contrast, in the non-elderly group (n = 8), three subjects had a T_max_ of approximately 1.93 min and five at 4.00 min. The shorter median T_max_ in the elderly cohort likely underlies their higher peak concentration (2,970 ng/mL vs. 1,330 ng/mL in non-elderly). Additionally, the ${\text{AUC}}_{0-\infty }$ was 23.54% higher in healthy elderly subjects (90% CI: 107.57%–141.87%). Although the upper bound of this confidence interval exceeds the standard bioequivalence threshold of 125%, the increased exposure does not compromise the clinical efficacy of ET-26. Some studies have indicated that brain sensitivity to etomidate remains constant with age. This may explain why the pharmacodynamic endpoints, such as the time to loss of consciousness, were similar between healthy elderly (1.82 min) and healthy non-elderly (1.93 min) subjects.

The safety profile of ET-26 was evaluated in both healthy elderly and healthy non-elderly subjects. In the healthy elderly cohort, drug-related TEAEs included injection site pain (12.5%) and cough (12.5%). Notably, the cough was effectively controlled through non-pharmacological interventions such as suctioning, and both the cough and the injection site pain resolved completely without further treatment. In contrast, in the healthy non-elderly group, the most frequently observed adverse events were injection site pain and myoclonus. These adverse events may be attributable to age-related physiological differences. Specifically, degenerative changes in the skin and peripheral nervous system among the elderly, such as reduced sensitivity of nociceptive nerve endings and slower nerve conduction velocity may account for the lower incidence of injection site pain in this group (Junker et al., 2023; Bozzetto Ambrosi and Bozzetto Ambrosi, 2025). Although elderly subjects showed slightly higher pharmacokinetic exposure compared to non-elderly subjects, this did not translate to increased safety risks, with no significant differences in safety profiles observed between the two groups. Overall, these findings indicate that ET-26 was well tolerated across both populations.

Several limitations should be acknowledged. The relatively small sample size and the exclusive inclusion of male, Han ethnicity subjects limit the generalizability of the findings. Moreover, as a single-dose study, the data do not capture the potential cumulative effects associated with repeated dosing, which are important for long-term clinical applications. Future studies should consider a more diverse subject population and explore the PK/PD profile under multiple dosing conditions. To build on these findings, further research is recommended to conduct larger-scale studies including both genders and subjects from diverse ethnic backgrounds.

In summary, a single intravenous administration of ET-26 at 0.8 mg/kg was generally well tolerated in both healthy elderly and non-elderly subjects. Although pharmacokinetic parameters differed significantly between the two groups—with the elderly demonstrating higher drug exposure—the efficacy endpoint, as measured by the time to loss of consciousness, was similar across both populations. Overall, these findings suggest that ET-26 possesses a favorable safety profile and comparable efficacy in diverse age groups despite different systemic exposure.

## Data Availability

The data underlying this article will be shared on reasonable request to the corresponding authors.
