# Supplementary material for: Comparative study of the pharmacokinetics, efficacy and safety of ET-26 in elderly and non-elderly subjects: the results of a phase I clinical trial
Source: Front Pharmacol. 2025 Oct 16;16:1665322. doi: 10.3389/fphar.2025.1665322 (PMC12573091; doi:10.3389/fphar.2025.1665322)
Supplement: Supplementary file 1 [file Supplementaryfile1.docx]

**Key inclusion/exclusion criteria**

Inclusion criteria

Subjects must meet all of the following inclusion criteria to be eligible for entry into this study:

1. Eligible subjects must be healthy adult males or females aged over 18 years.
2. Subjects must have a body mass index (BMI) between 18.0 and 30.0 kg/m² (inclusive). Additionally, male subjects must weigh at least 50 kg, and female subjects must weigh at least 45 kg.
3. Subjects should have blood pressure within the range of 90-139 mmHg systolic and 60-89 mmHg diastolic, with heart rate and pulse between 55-100 beats per minute.
4. Subjects must be able to communicate effectively with the investigator, voluntarily sign the informed consent form, and comply with the protocol requirements to complete the study.

Exclusion Criteria

Subjects would not participate in this study if they have any of the following characteristics or conditions:

*Supplementary Examinations:*

1. Abnormal results in physical examination, vital signs, 12-lead electrocardiogram, cortisol test, and laboratory tests (complete blood count, blood biochemistry, coagulation function, urinalysis) that are considered clinically significant by the investigator.
2. Potential difficult airway (airway assessment with a modified Mallampati score of III-IV).
3. Positive for hepatitis B surface antigen, hepatitis C antibody, HIV antibody, or syphilis antibody.

*Medication History:*

1. Use of any drug that inhibits or induces hepatic drug-metabolizing enzymes within 30 days before screening.
2. Use of any prescription medication within 14 days before dosing.
3. Use of over-the-counter drugs, herbal medicine, or dietary supplements (e.g., vitamins, calcium supplements) within 7 days before dosing.

*Medical and Surgical History:*

1. History of any clinically significant disease or condition that, in the investigator's judgment, may interfere with the trial results, including but not limited to conditions of the cardiovascular, respiratory, endocrine, nervous, digestive, urinary, blood, immune, psychiatric, or metabolic systems.
2. History of adrenal insufficiency, adrenal tumor, or hereditary hemoglobin biosynthesis disorder.
3. Any surgery within 6 months before screening.
4. Known allergy history to two or more substances or in the investigator's judgment, a potential for allergic reactions to the investigational drug or its excipients.

*Lifestyle Habits:*

1. Alcohol abuse or regular alcohol consumption within 6 months before screening, i.e., drinking more than 14 units of alcohol per week (1 unit = 360 mL of beer, 45 mL of 40% alcohol spirits, or 150 mL of wine); or positive alcohol breath test at baseline.
2. Smoking more than 5 cigarettes per day on average in the 3 months before screening, or inability to refrain from smoking during the trial.
3. History of drug abuse or illicit drug use in the past 3 months; or positive urine drug test at baseline.
4. Habitual consumption of grapefruit juice or excessive tea, coffee, and/or caffeinated beverages, and inability to abstain during the trial period.

*Others*

1. Individuals with difficulties in blood collection, inability to tolerate venipuncture, or arterial blood sampling (e.g., positive Allen's test, applicable only to Series A and Series B).
2. Participants who have been involved in any other clinical trials within the last 3 months, including trials involving drugs and medical devices.
3. Individuals who have received vaccinations within the last month or plan to receive vaccinations during the trial period.
4. Pregnant or nursing women.
5. Participants who plan to conceive or donate sperm during the trial period and for six months after the trial, or those who do not agree to practice strict contraceptive measures during the trial period and for six months after the trial.
6. Individuals who have experienced blood loss or donated blood exceeding 400 mL within the last 3 months, or those who have received a blood transfusion within the last month.
7. Subjects deemed by the investigator to have any factors that make them unsuitable for participation in this trial.

**Ethical study conduct**

The clinical study protocol and the informed consent form as well as their amendments had been reviewed and approved by the Ethics Committee of the Shandong Provincial Qianfoshan Hospital before implementation (Ethical approval number: 2023302). The composition of the ethics committee complied with the requirements of the National Medical Products Administration (NMPA) and the ethics committee fulfilled all the required responsibilities. Before the initiation of the study, the investigator and the sponsor had submitted to the ethics committee all study-related documents and materials required by the ethics committee following applicable regulations and obtained written approval opinions from the ethics committee on the study protocol, informed consent form, and procedures as well as any other written information to be provided to the subjects that need to be signed and dated. During the study, both the investigator and the sponsor submitted reports, data updates, and other information to the ethics committee as required or as per institutional procedures. This study was designed, supervised, and conducted following the regulatory requirements of the Declaration of Helsinki (2013) "Ethical Principles for Human Medical Research", ICH GCP E6 (R2), and "Good Clinical Practice" issued by NMPA and as per the sponsor's standard operating procedures (SOPs).

**Bioanalytical Method for Plasma Sample Determination**

For all subjects, venous blood was drawn at specified time points, including within 1 hour before administration (-1 to 0 h), immediately after administration (0 h), and at 1 min, 3 min, 5 min, 10 min, 15 min, 30 min, 1 h, 2 h, 4 h, 8 h, and 24 h post-administration. The collected blood samples were transferred into collection vessels containing NaF-EDTA-K2 as an anticoagulant. Plasma was then separated by centrifugation and primarily used for pharmacokinetic analysis. At 1 minute and 4 hours after drug administration, collect 5 mL of venous blood into blood collection tubes containing NaF-EDTA-K2 anticoagulant. Centrifuge the samples to obtain plasma for determination of plasma protein binding rate. The liquid chromatography-tandem mass spectrometry (LC-MS/MS) was used to quantify the plasma concentrations of ET-26. The chromatographic conditions are detailed in Table S1. The lower limit of quantification (LLOQ) and calibration ranges were 10.00 ng/mL (10.00-4000 ng/mL) for ET-26. The accuracy and precision for all analytes were within ±15%.

Table S1. Overview of the Analytical Method

| **pound** | **Methoxyethyl etomidate (ET-26)** | |
| --- | --- | --- |
| **LC System** | Shimadzu HPLC-20 AD | |
| **Column** | YMC-Triart C18, 50×2.0 mm | |
| **Column Temperature** | 40°C | |
| **Auto Injector Temperature** | 10°C | |
| **Mobile Phase** | A: Salt Solution – Ultra Pure Water (1:1000, v/v)  B: Acetonitrile | |
| **Flow Rate** | 0.4 mL/min | |
| **Mass Spectrometer** | Applied Biosystems API 4400 & 4000 QTRAP | |
| **Ionization** | Positive ESI | |
| **Ion Pairs (m/z)** | Methoxyethyl etomidate (ET-26) | ET-26 Deuterated Compound (IS) |
|  | Parent Ion: 275.3 /  Fragment Ion: 171.1 | Parent Ion: 278.1 /  Fragment Ion: 172.2 |
| **Declustering Potential (DP)** | 40 | 40 |
| **Entrance Potential（EP）** | 13 | 10 |
| **Collision Energy（CE）** | 20 | 10 |
| **Cell Exit Potential（CXP）** | 8 | 15 |

Table S2. Detailed criteria for adverse events.

| Grading | Severity Description |
| --- | --- |
| Grade 1 | Mild; asymptomatic or mild symptoms; clinical or diagnostic tests only; intervention not indicated. |
| Grade 2 | Moderate; minimal, local, or noninvasive intervention indicated; limiting age-appropriate activities of daily living (e.g., preparing meals, shopping or buying clothes, using the telephone, managing money, etc.). |
| Grade 3 | Severe or clinically significant but not immediately life-threatening; hospitalization or prolongation of hospitalization indicated; disabling; limiting self-care ability (refers to the ability to bathe, dress and undress, eat, go to the toilet, take medications, and be bedridden). |
| Grade 4 | Life-threatening consequences; urgent medical intervention indicated. |
| Grade 5 | Death related to AE. |

Mild: Grade 1; Moderate: Grade 2; Severe: Grade 3 or higher.

*The severity of adverse events was classified according to the standardized criteria outlined in the National Cancer Institute's Common Terminology Criteria for Adverse Events (CTCAE v5.0).*
